# Dual AAV/IL-10 Plus STAT3 Anti-Inflammatory Gene Delivery Lowers Atherosclerosis in LDLR KO Mice, but without Increased Benefit

**DOI:** 10.1155/2012/524235

**Published:** 2011-09-11

**Authors:** Maohua Cao, Junaid A. Khan, Bum-Yong Kang, Jawahar L. Mehta, Paul L. Hermonat

**Affiliations:** ^1^Central Arkansas Veterans Healthcare System, 111J, 4300 West 7th Street, Little Rock, AR 72205, USA; ^2^Research of Cardiology, University of Arkansas for Medical Sciences and VA Medical Center, Little Rock, AR 72205, USA; ^3^Department of Medicine, Atlanta Veterans Affairs and Emory University Medical Centers, Atlanta, GA 30033, USA

## Abstract

Both IL-10 and STAT3 are in the same signal transduction pathway, with IL-10-bound IL10 receptor (R) acting through STAT3 for anti-inflammatory effect. To investigate possible therapeutic synergism, we delivered both full-length wild-type human (h) STAT3 and hIL-10 genes by separate adenoassociated virus type 8 (AAV8) tail vein injection into LDLR KO on HCD. Compared to control Neo gene-treated animals, individual hSTAT3 and hIL-10 delivery resulted in significant reduction in atherogenesis, as determined by larger aortic lumen size, thinner aortic wall thickness, and lower blood velocity (all statistically significant). However, dual hSTAT3/hIL-10 delivery offered no improvement in therapeutic effect. Plasma cholesterol levels in dual hSTAT3/hIL-10-treated animals were statistically higher compared to hIL-10 alone. While no advantage was seen in this case, we consider that the dual gene approach has intrinsic merit, but properly chosen partnered genes must be used.

## 1. Introduction

Animal and humans studies have lead to at least a partially understanding of the role of inflammatory cells in atherogenesis [1]. From this understanding we now know that atherosclerotic plaque is essentially a benign immune/inflammatory cell tumor. A cascade of events is believed to result in the development of such plaque. Endothelial cells become activated by shear stress or other insults. These activated endothelial cells then stimulate trafficking of immune cells into the intimal area, most notably monocytes. Monocytes are the precursors of macrophages and, ultimately, of lipid engorged macrophages, known as foam cells. These foam cells ultimately form the major mass of early intermediate atherosclerotic plaque. Inflammatory cytokines are overexpressed in this environment, changing the arterial lumen milieu from an antithrombogenic state to a prothrombotic one. In this altered milieu activated endothelium displays decreased nitric oxide synthesis and an increased oxidative state, which, in turn, increases inflammation [2, 3].

There are now a variety of anti-inflammatory genes already identified which might be used with therapeutic effect. Cytokine interleukin 10 (IL-10) has been significantly studied as a strong general inhibitor of immune response and inflammation. In mouse models IL-10 gene delivery does result in moderate antiatherosclerotic effects, shown by multiple groups [4–6]. It is also known that IL-10 signaling goes through, requires, signal transducer and activator of transcription 3 (STAT3) [7, 8] and that STAT3 gene delivery also results in inhibition of atherosclerotic plaque formation [9]. Yet, while individual IL-10 and STAT3 gene delivery were efficacious, some disease remained. Thus, yet stronger gene effectors are desirable. This issue is particularly important considering that the most likely candidates for antiatherosclerosis gene therapy will have significant established disease. 

In the field of cancer it is well known that multiple protooncogene mutations often take place within a single signal transduction pathway, resulting in continuous and permanent signaling within those malignant cells, most commonly signaling continuous cell division [10]. This is a powerful form of functional gene cooperation which ultimately results in many cancer deaths. We considered this cancer-causing gene signaling cooperation to have a powerful phenotype and that this strategy could potentially be useful for addressing atherogenesis. We hypothesized that perhaps the overexpression of two genes within a common signal transduction pathway, using anti-inflammatory genes in place of oncogenes, may result in a beneficial gene cooperation and provide an even stronger anti-inflammatory effect. Moreover, this is a novel gene therapy approach, not attempted before. 

We have previously studied the individual use of the IL-10 and STAT3 genes to lower inflammation and atherogenesis [4, 9]. These two genes are located within the same anti-inflammatory signal transduction network, with IL-10 acting through STAT3 [7, 8]. To test the hypothesis that two anti-inflammatory genes of the same pathway will have higher efficacy than one, hIL-10 plus hSTAT3 was delivered using adenoassociated virus (AAV) as a gene delivery vector [4, 9], and the resulting therapeutic effect was studied in a LDLR KO mouse-HCD model. AAV is an outstanding gene therapy/gene delivery vector, having been used since 1984 [11–13], and does not contribute to inflammation [14]. Contrary to our thoughtful planning, no increased therapeutic benefit was observed when using STAT3/IL-10 together, and, in fact, cholesterol levels were higher in the dual-gene-treated animals.

## 2. Methods

### 2.1. Generation of Recombinant AAV Virus

Construction and generation of AAV/Neo, AAV/hSTAT3, and AAV/hIL-10 recombinant virus have been described previously [4, 7, 9]. The virus stocks were generated and tittered by dot blot hybridization as described previously [4, 7, 9]. The titers were calculated to be about 1 × 10^9^ encapsidated genomes per mL (eg/mL).

### 2.2. Animal Treatments

Low-density lipoprotein receptor (LDLR) knockout mice (B6;129S7-*Ldl*r^tm1Her^/J) were obtained from Jackson Laboratories (Bar Harbor, Me, USA). Two groups of male mice weighing 16–20 grams were injected with AAV-Neo and AAV-STAT3 virus each at a titer of 1 × 10^9^ eg/mL via tail vein injections of 200 *μ*l virus/mouse, followed by two booster injections at an interval of less than one week. The animals were started on high cholesterol diet (HCD) of 4% cholesterol and 10% Coco butter diet (Harlan Teklad, Madison, Wis, USA) on the day of first injection and maintained for twenty weeks. This HCD was used to ensure the development of atherosclerosis. Mice fed normal chow diet were included as experimental control group. Animals were weighed weekly, and all experimental procedures were performed in accordance with protocols approved by the Institutional Animal Care and Usage Committee of the Central Arkansas Veterans Health Care System at Little Rock.

### 2.3. High-Resolution Ultrasound Imaging

Ultrasound imaging was done using the Vevo 770 High-Resolution Imaging system (Visualsonics, Toronto, Canada) with a RMV 707B transducer having a center frequency of 30 MHz. Animal preparation was done as described earlier [15]. Briefly, each mouse was anesthetized using 1.5% isoflurane (Isothesia, Abbot Laboratories, Chicago, USA) with oxygen and laid supine on a thermostatically heated platform with all legs taped to ECG electrodes for cardiac function monitoring. Abdominal hair was removed with a shaver and a chemical hair remover (Church & Dwight Co., Inc., NJ, USA), and a prewarmed US gel (Medline Industries, Inc., Mundelein, USA) was spread over the skin as a coupling medium for the transducer. Two levels of the vessel were visualized: thoracic region—below the aortic arches to the diaphragm and the renal region—the upper abdominal region to the iliac bifurcation. Image acquisition was started on B-mode, where a long-axis view was used to visualize the length of the aorta. Next, the scanhead probe was turned 90° for a short-axis view to visualize the cross-sectional area of the aorta. Individual frames and cine loops (300 frames) were acquired at all levels of the aorta both in long-axis and short-axis view and recorded at distances of 1 mm throughout the length of the aorta. For measurement of flow velocity, orientation of the abdominal aorta on ultrasound was accomplished by tilting the platform and the head of mouse down with the transducer probe towards the feet and tail of the mouse. This positioning ensured the Doppler angle to be less than 60°  for accurate measurements of blood flow velocity in the pulse wave Doppler (PW) mode within abdominal aorta. Measurements and data analysis was performed off line using the customized version of Vevo 770 Analytical Software from both the longitudinal and transverse images. The complete imaging for each mouse lasted for about 25–30 minutes.

### 2.4. Measurement of Plasma Cholesterol

Plasma levels of total cholesterol for AAV8/hSTAT3 and AAV8/Neo mice were measured by VetScan VS2 (ABAXIS, Union City, Calif) at the Veterans Animal Laboratory (VAMU).

### 2.5. hSTAT3 and IL-10 Gene Expression Analysis Using Real-Time Quantitative Reverse Transcription PCR (qRT-PCR)

Total RNA from aorta of three mice was extracted with TRIzol extraction (Invitrogen Carlsbad, Calif) according to the manufacturer's instructions. cDNA was synthesized using random hexamer primers and RNase H-reverse transcriptase (Invitrogen, Carlsbad, Calif). QRT-PCR was performed using the Applied Biosystems Fast 7900HT real-time PCR system (Applied Biosystems, Foster City, Calif) as described in [9]. We designed qRT-PCR specific primers for analyzing hSTAT3 and IL-10 using Probe-Finder (http://www.roche-applied-science.com) web-based software from Human and Mouse Universal ProbeLibrary from Roche Applied Science. The results were analyzed using SDS 2.3 relative quantification (RQ) manager software. The comparative threshold cycles (Ct) values were normalized for GAPDH reference genes and compared with a calibrator by the 2^−ΔΔCt^ method.

## 3. Results

### 3.1. AAV8 Delivers hIL-10 and hSTAT3

**Figure 1:**
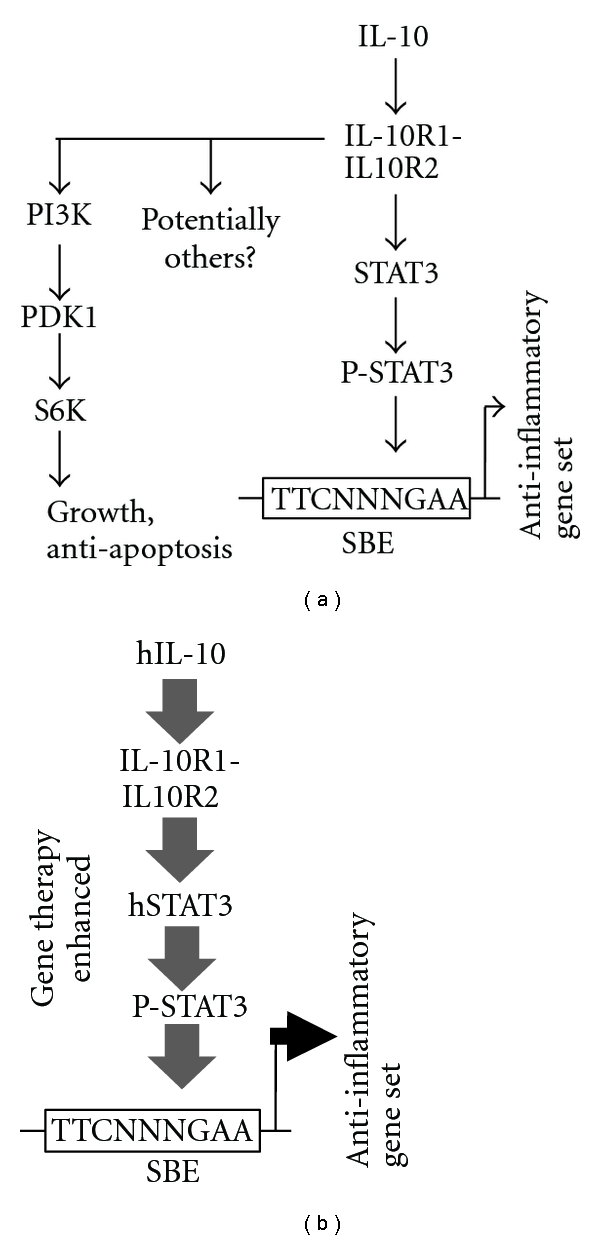
IL-10 signal transduction and hypothesized treatment by dual gene therapy. (a) shows multiple possible IL-10 signal transduction pathways. The STAT3 pathway results in an anti-inflammatory response. (b) shows our attempt to enhance IL-10 signaling at two points with the hypothesis that enhanced gene expression of the anti-inflammatory gene set would occur. The gene delivery is indicated by larger font and bolded IL-10 and STAT3. More powerful signal transduction is represented by larger arrows and larger font.

**Figure 2:**
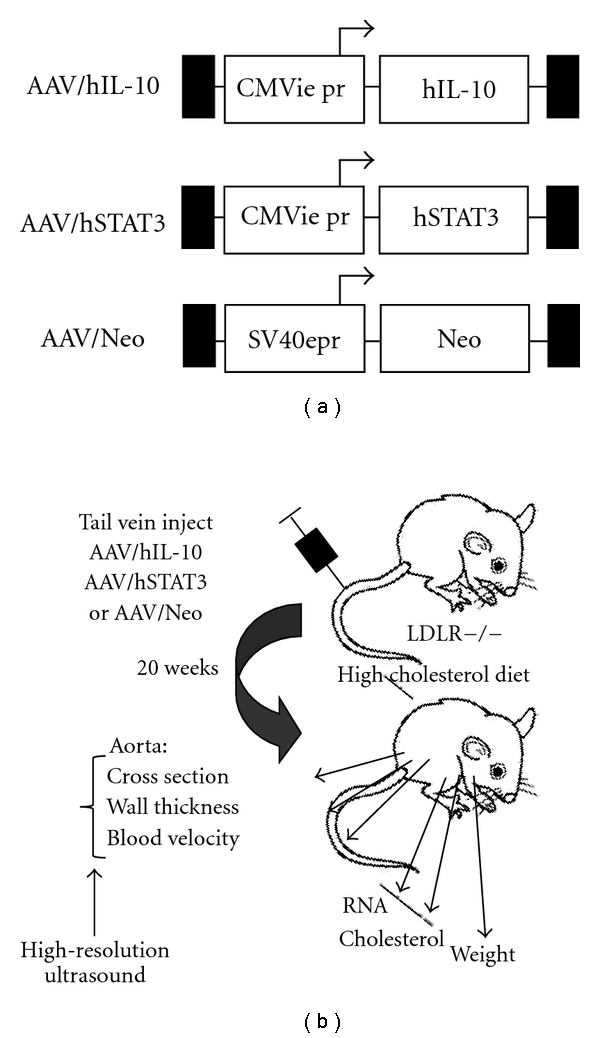
Structure of AAV vectors and experimental scheme. (a) shows the basic structure of the three AAV vectors used in this study. (b) shows the experimental scheme and data collected.

**Figure 3:**
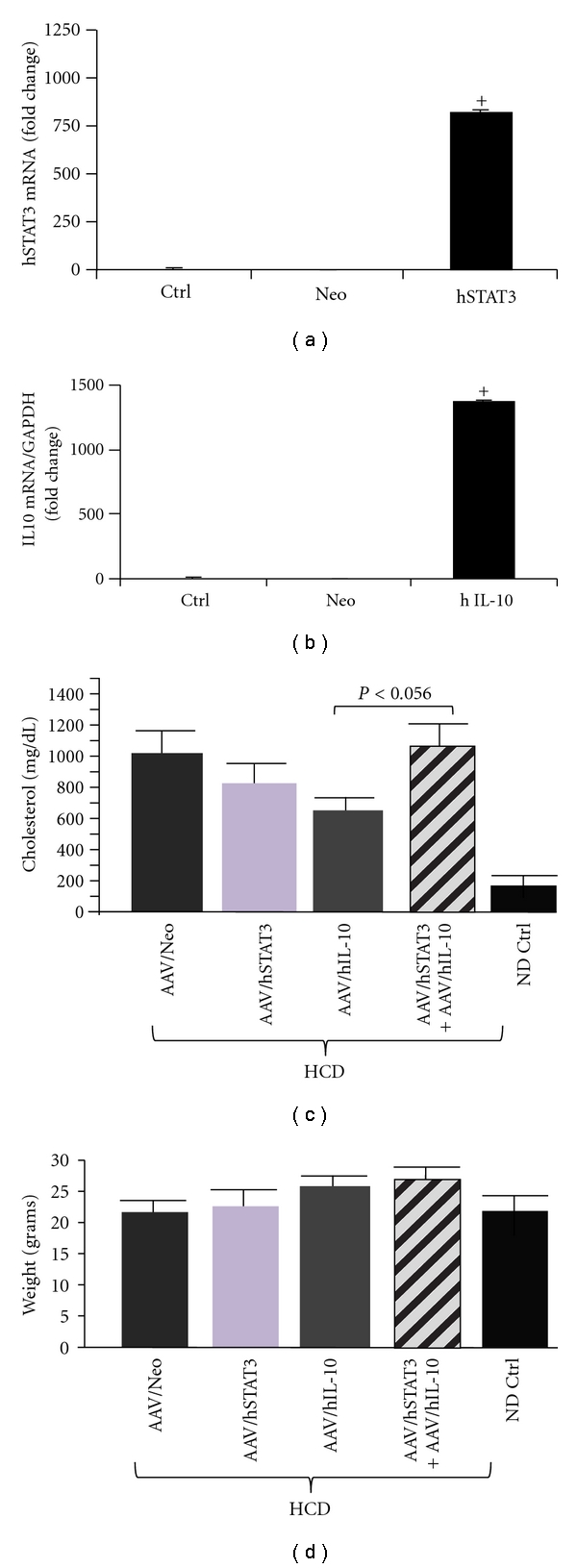
Expression of the delivered hSTAT3 and IL-10. Relative expression of hSTAT3 (a) and IL-10 (b) genes by real-time quantitative PCR from aorta of 3 mice in each group. For qRT-PCR the quantity of RNA for each gene was normalized to GAPDH in the same sample. Data shown are mean ± SE. (c) shows the levels of total cholesterol. Note that cholesterol levels of the hSTAT3-plus-hIL-10-treated animals had significantly higher cholesterol levels than animals treated with individual genes. (d) shows the animal weights at the end of the experiment. The key at the bottom is used for both panels (c) and (d).

Both IL-10 and STAT3 have been shown to inhibit atherosclerosis in an animal model [4–6, 9], and both are in the same anti-inflammatory signal transduction pathway, with IL-10 acting through STAT3 [7, 8]. Because of this we reasoned that the delivery of both genes together may result in a synergistic higher level of anti-inflammatory activity, which is depicted in Figure 1. To test this hypothesis and the efficacy of IL-10-plus-STAT-3 dual gene delivery we delivered both into LDLR KO mice using AAV8 and placed them on high cholesterol diet (HCD). An AAV/Neomycin resistance gene (Neo) vector was also used as a null, nontherapeutic control. Vector structures are shown in Figure 2(a) and the overall experimental scheme in Figure 2(b). Upon time of harvest, a portion of mice were sacrificed to determine the success of gene delivery by analyzing hSTAT3 mRNA expression in the aorta using qRT-PCR analysis. This analysis utilized mRNA isolated from 3 mice aortas from each group, harvested at week 20. Representative results for hSTAT3 and hIL-10 are shown in Figure 3(a), and both were observed to be highly expressed in aortas of appropriately treated animals but not AAV/Neo-injected or control animals.

### 3.2. Therapeutic hSTAT3, hIL-10, or hSTAT3-Plus-hIL-10 Gene Delivery Inhibits Aortic Blood Flow Velocity with Equal Efficacy

Following demonstration of successful transgene delivery studied the effects of the transgene. Figure 3(b) shows that hSTAT3-treated animals had total cholesterol levels comparable to Neo-treated HCD-fed animals; however hIL-10-treated animals were statistically lower. Yet it was found that the dual hIL-10-plus-hSTAT3-treated animals had cholesterol levels which trended higher than either hIL-10- or hSTAT3-treated animals. Regarding animal weights, all treatments were statistically the same as normal diet (ND) controls but trended higher. 

**Figure 4:**
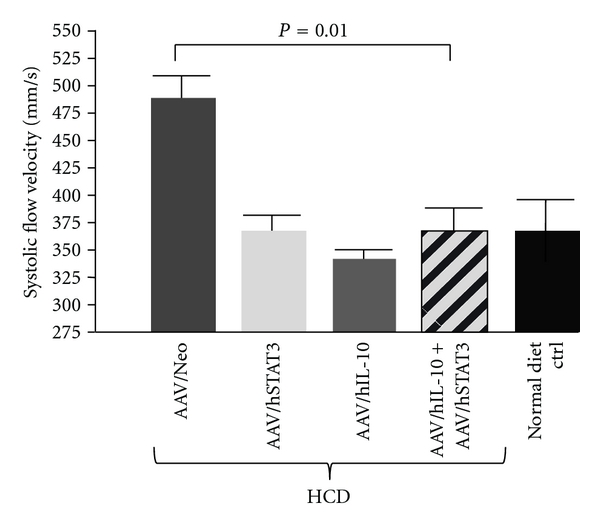
Systolic blood velocity. High-resolution ultrasound (HRUS) was used to measure blood flow velocities in the lumenal center of the abdominal region of the aorta in 3–5 animals from each group. Shown is a quantification of the results. Note that the hSTAT3-treated, the hIL-10-treated, and the hSTAT3-plus-hIL-10-treated animals all had significantly lower blood velocity than the AAV/Neo animals.

We utilized blood flow velocity measurement as a novel technique to quantify atherosclerosis in the mice. Systolic blood flow velocity in the lower abdominal region of the aorta was quantified by high-resolution ultrasound (HRUS) imaging system Vevo 770 with measurements taken on three to five animals. Figure 4 shows the quantified the systolic blood velocity from five separate measurements on each animal. As shown, the AAV/Neo-HCD-treated animals, with the highest lipid deposition, displayed the highest flow velocity. Moreover, all three therapeutic treatments had markedly lower flow velocity, very similar to that in normal-diet-fed control animals. Thus all three treatments showed antiatherosclerotic efficacy, yet the dual gene treatment was not statistically improved over the individual gene treatments, nor did dual gene delivery trend towards higher efficacy.

### 3.3. Therapeutic hSTAT3, hIL-10, and hSTAT3-Plus-hIL-10 Gene Delivery Inhibits Aortic Structural Changes Associated with Atherosclerosis with Equal Efficacy

**Figure 5:**
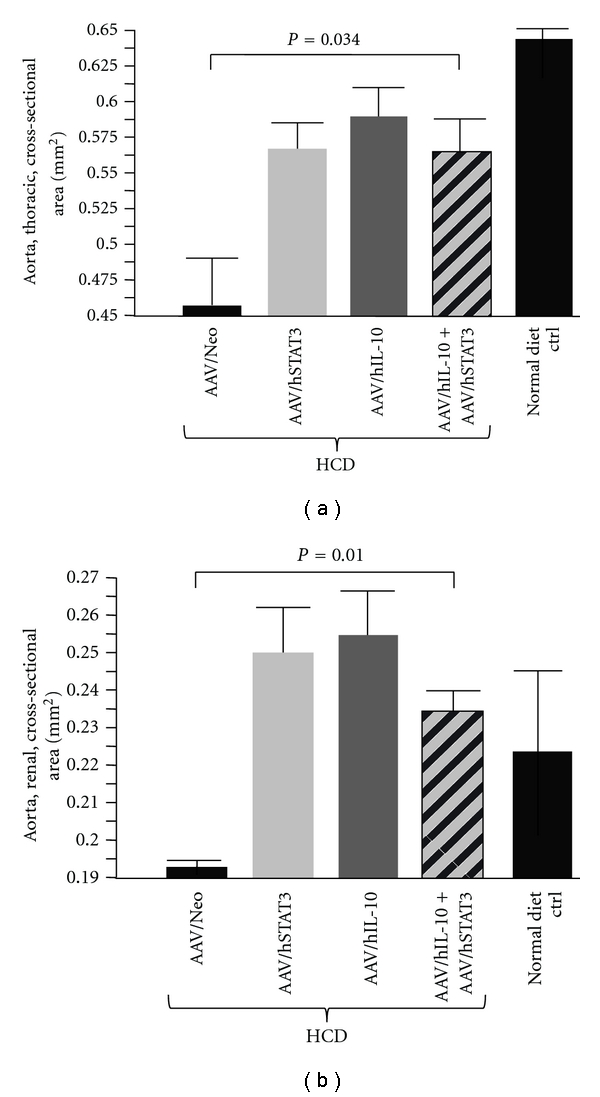
Analysis of the aortic lumen by high-resolution ultrasound (HRUS). HRUS was used to measure the cross-sectional area of the thoracic and renal regions of the aortas in 3–5 animals from each animal group. (a) shows quantification of the cross-sectional area for the thoracic region of the aorta. Note that the hSTAT3-treated, the hIL-10-treated, and the hSTAT3-plus-hIL-10-treated animals all had a much larger cross sectional area than the AAV/Neo-treated animals, indicating significant efficacy. Similarly in (b), quantification of the data for the renal region of the aorta shows a much larger lumen size for the hSTAT3-, hIL-10-, and hSTAT3-plus-hIL-10-treated animals compared to AAV/Neo-treated animals.

Next structural changes in the aortas resulting from the various treatments were quantified using HRUS imaging. Multiple measurements were made in three to five animals from each group and compared. The measurements were made in the same indicated site (see Section 2). The cross-sectional area of the lumen of the thoracic region of the aorta was one measurement taken, with five readings from each animal. Figure 5(a) shows the quantified results for the thoracic region of the aorta. The AAV/Neo-HCD-treated positive control animals displayed the smallest cross sectional lumen area, consistent with significant atherosclerosis. In contrast, all three therapeutic treatments (hSTAT3, hIL-10 or hSTAT3-plus-hIL-10) had much larger lumens than Neo-treated-HCD controls, and this difference was statistically significant. Normal diet control animals had the largest lumen. The renal region of the aorta showed a similar pattern to the thoracic aorta, as shown in Figure 5(b), with AAV/Neo-HCD-treated having the smallest and all three therapeutic treatments having statistically significant larger lumens. Thus, again, all three treatments showed antiatherosclerotic efficacy, yet the dual gene treatment was not statistically improved over the individual gene treatments. 

**Figure 6:**
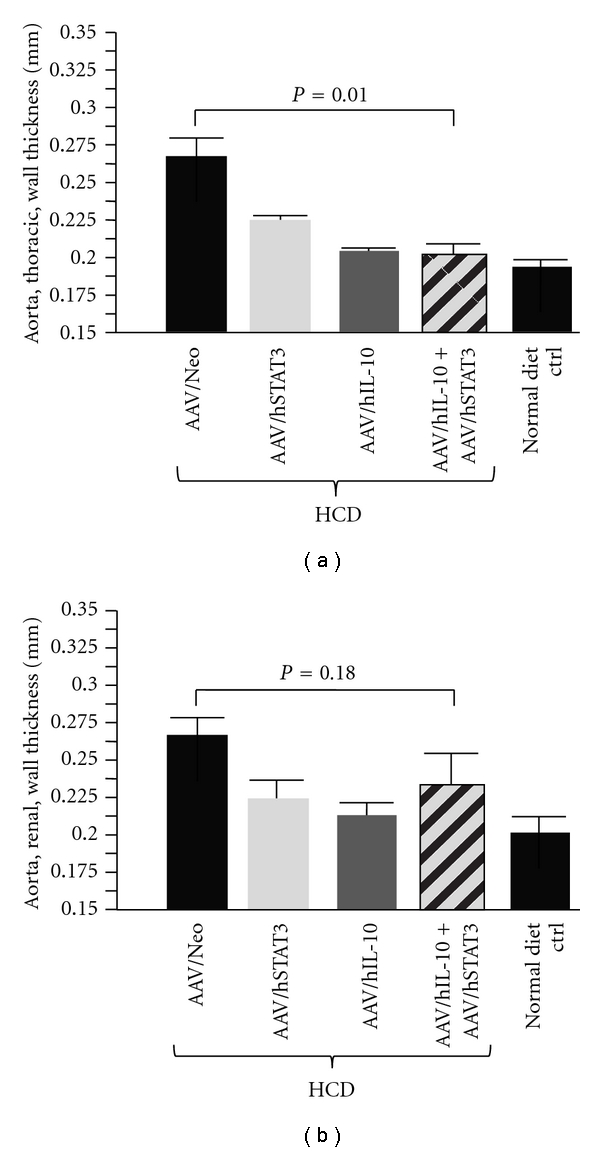
Analysis of the aortic wall thickness by HRUS. HRUS was used to measure the wall thickness of the aorta. (a) shows quanitification of the thoracic region of the aortas in 3–5 animals from each animal group. Note that the hSTAT3-, hIL-10-, and hSTAT3-plus-hIL-10-treated animals had thinner wall thicknesses than the Neo-treated animals, indicating significant efficacy. (b) shows quantification of the renal region of the aortas in 3–5 animals from each animal group.

The wall thickness of the thoracic region of the aorta was yet another measurement made, with data from three to five animals and five readings from each animal. Figure 6 shows the quantified results for the thoracic region of the aorta. The AAV/Neo-treated animals displayed the thickest thoracic walls, and all three therapeutic treatments gave statistically thinner walls, (*P* = 0.05). Yet, again, dual gene delivery failed to improve therapeutic efficacy.

## 4. Discussion

This study is the first attempt at using dual gene therapy within a single signal transduction pathway with a believed beneficial phenotype with the hypothesis that improved efficacy will result. Both IL-10 and STAT3 are anti-inflammatory genes within the same signal transduction pathway. Because of this we hypothesized that a synergistic anti-inflammatory effect or enhanced therapeutic effect might be observed by both AAV8/hIL-10 plus AAV8/hSTAT3 dual gene delivery in LDLR KO mouse on HCD. This study demonstrates that there was no enhanced efficacy when both genes were delivered together over that of the individual genes. One possible explanation for this lack of effect is that the two genes were delivered by separate AAV vectors and thus likely that very few cells were receiving and actively expressing both transgenes. If the proteins of both genes were intracellular then this explanation would likely be correct and fully prevent gene cooperation. However, while hSTAT3 is intracellular, IL-10 is a secreted protein and its activity should be effective over an area through its diffusion. Thus, we think that this explanation is not a viable mechanism as to why there is no additive or synergistic therapeutic effect by this approach. Another possible contributor to the lack of enhancement is that IL-10 trends to have a greater effect than STAT3; thus what we may be viewing is only the dominant IL-10 effect. Alternatively, the lack of improved efficacy by dual IL-10/STAT3 delivery may also suggest that another IL-10 pathway is more effective in inhibiting inflammation than the STAT3 pathway. Yet enhancement of the PI3K pathway would seem inappropriate as PI3k expression appears positively associated with atherosclerosis [16].

Cholesterol levels seen in the dual-gene-treated animals were higher than in either individual gene-treated animals (statistically significant for hIL-10 versus hIL-10-plus-hSTAT3). As both IL-10 and STAT3 are individually associated with lower cholesterol levels [5, 6, 9], these data may suggest that some type of negative regulatory interference between these two gene's functions and their downstream signaling is taking place. Additionally, their individual mechanisms for lowering cholesterol may also be abrogated. The mechanism of how each lowers blood cholesterol levels is presently unclear. 

Yet another possibility for the failure of IL-10 and STAT3 to cooperate may be the “crossroads” position of STAT3 in the transduction of signaling from multiple receptors, beyond IL-10/IL-10R interaction. Other important signaling pathways which require STAT3 signaling include IL-6 [17], IL-17 [18], Ang II [19], and thrombin [20]. While it is unclear which of these pathways (or others) is active and might serve to inhibit the IL-10-STAT3 signaling pathway, this study reminds us of the complexity of cellular signaling and the need to determine signaling pathway dominance or interference when medically significant issues of IL-10 and STAT3 are at issue. An alternative gene pair which might be tried would be IL-10 plus IL10R1. This would concentrate augmentation at only the ligand-receptor level, and the complexities of STAT3 overexpression might be avoided. 

It is surprising that IL-10 and STAT3 overexpression should apparently “knock out” each other's cholesterol regulation phenotype as well as limit each other's antiatherogenesis phenotype. Due to our negative results we did not investigate the failure of cooperation between IL-10 and STAT3 any further. Pursuing the mechanism of action of negative data is unjustified. However, these results of noncooperation indicate that our understanding of the IL-10 and STAT3 pathways is incomplete and that gene pairs utilized in dual gene therapies may give surprising results different than what initial logical planning might suggest. However, we believe the dual gene delivery approach will ultimately result in enhanced efficacy if the gene partners chosen are mechanistically appropriate and compatible. In spite of the negative results presented here, the investigation and discovery therapeutic gene synergisms, but not between IL-10 and STAT3, has merit and needs to be pursued.
